# An Ancient Practice but a New Paradigm: Personal Choice for the Age to Spay or Neuter a Dog

**DOI:** 10.3389/fvets.2021.603257

**Published:** 2021-03-19

**Authors:** Lynette A. Hart, Benjamin L. Hart

**Affiliations:** School of Veterinary Medicine, University of California, Davis, Davis, CA, United States

**Keywords:** castration, humans, livestock, luteinizing hormone, breed differences, sex differences, body size, gonadal hormones

## Abstract

Extensive practice and knowledge of the methods and effects of castration of male livestock and even humans has been widespread since ancient times, but only a few decades ago did neutering (including spaying) become a routine part of canine husbandry. In the US, the 6-month neuter became standard practice. Only recently, however, have some of the consequences of this major physiological alteration become evident. As the data-based study on 35 breeds reveals, there are major breed differences associated with effects of neutering, especially with early neutering, including increased risks of joint disorders and some cancers. The study of mixed-breed dogs reveals that the risk of joint disorders is increased in the large dogs. Implications of breed-specific and sex-specific effects for age of neutering have prompted the consideration of a new paradigm with regard to this practice. This involves focusing on each individual dog when deciding upon the appropriate age of neutering to avoid increasing the risk of a joint disorder or cancer above that inherent for the breed. For many breeds, particularly the smaller dogs, no effects were found for the age of neutering on joint disorders and the cancers followed. In these cases, the caregiver has a wide range of choice for neutering without increasing the dog's risk for these diseases. In the future, additional research may reveal more about other increased risks for age-related cognitive dysfunction or elevated levels of luteinizing hormone caused by gonad removal, and lead to revised guidelines.

## Early Practices of Neutering and Spaying Animals and Castrating Humans

Having dogs as companions to humans dates back to prehistoric times: recent archeological studies of dental microwear of canine skeletons suggest that early protocols differed in their diets from wolves, were behaviorally and morphologically distinct, and already were companions to humans in the Paleolithic age, over 28,000 years ago (1).

As farming by early humans came to include husbandry of cattle, horses, sheep, and goats, castration of male livestock animals became commonplace, and was practiced at least 8,000 years ago (2). Gonadectomized male livestock were strong enough for doing work and easier to manage than unaltered bulls; these oxen, being castrated, were the favored draft working animals. Interestingly, the ancient Sumerians passed laws around 2400 BC protecting the welfare and affirming the value of their oxen, specifying penalties for injuries to oxen when they were rented (3). An injury to the oxen's horn had a penalty of one-third of the oxen's value, to the eye was one-half the value, and if drowned, the full value or a replacement ox was to be paid. Similarly, King Hammurabi of Babylon around 1750 BC passed 29 law codes concerning crimes against oxen and specifying rules regarding payments by someone hiring oxen or for veterinary bills (4). Breaking off a horn, cutting of a tail, or hurting its muzzle required paying one-fourth of its value in money to the owner. A veterinarian performing a serious operation on an ox and killing it, was required to pay the owner one-fourth of its value. Someone stealing an ox needed to pay back thirty times its value (5).

Castrating male livestock, a relatively easy procedure, was commonly practiced in Egypt (6). Ancient Egyptians maintained huge herds of cattle, sheep, and goats; for example, over 400,000 livestock were maintained for the temple at Thebes (1, p. 43). The cattle selected for use in special religious ceremonies there invariably were castrated bulls (6).

In ancient Greece, most cattle, sheep, goats, and pigs were castrated, except for a few males needed for breeding (7). As well-known then, the castration reduced problems with aggression in groups of animals, and the castrated males yielded more and fattier meat. Galen explained that oxen were castrated for usefulness in farming, and pigs to yield more tasty meat (6). With swine, the meat of a castrated male tasted better than that from intact males and thus provided a way to avoid the “boar taint.” Neutering of animals in ancient Greece is also depicted in engravings on urns and jugs (7). During wars, oxen, mules, and asses marched with the army, carrying heavy loads (8). Large flocks of sheep, primarily castrated males, were raised for wool (9). Aristotle advocated that all authors should possess knowledge on a variety of topics, including the castration of a boar (8). Although many male animals were castrated, ancient documents from Greece around 200 BC provide evidence that at the same time, the uncastrated male animals represented a cultural ideal that was preferred for sacrifice and rituals (7).

In Asia Minor, castration sometimes was incorporated into religious or sacrifice ceremonies. Around the world and throughout ages, people were making decisions on specifically which males to neuter and which method to use, while also choosing not to castrate certain individuals. Spaying females, or ovariohysterectomy, did not come into practice until much later, undoubtedly because of the greater surgical complexity and risks of infections. Even now, spaying females is not common in the husbandry of livestock or horses.

The prevalent practice in major ancient cultures of castrating boys and men was a practice that produced eunuchs who had special roles in several ancient societies; awareness of the methods, purposes, and effects of human castration was inevitable. Studies of eunuchs in Late Antiquity and the court cultures of Byzantium, Islam, and China are available, outlining their important integrated roles in society; less is known about the worldwide slave trade in eunuchs in the Afro-Euro-Asian world (10, 11). Also, the Bible contains numerous references to eunuchs (12). Some men castrated themselves for religious reasons to acquire improved status in the church (13). Others were castrated for a variety of reasons, with different outcomes. Well known is the castration of young boys as Roman castrati singers from third century BC through sixth century AD (14). Considering the ubiquity of human castration in the ancient world, a somewhat sophisticated awareness of castration must have resulted. The methods and employment of castration of humans, as with animals, involved specific decisions in each situation.

In recent times, castrated cattle were selected to be the special display bulls of the Dinka people, as was done in ancient Egypt, and they also would be the ones selected for sacrifice (15). The Dinka often delayed the castration of bulls and rams until the animals were a bit older, presumably to allow the conformation and characteristics of the animal to be manifest before making a decision on whether to castrate. Most bulls were castrated for important cultural and esthetic reasons, to reduce fighting, for easier control, and to prevent indiscriminate breeding of estrous cows. Little is known about castration of dogs and cats during the Renaissance, though a wall painting depicted castration of a cat in 1531–1532 (16).

In the twentieth century, when many animals were living on farms, people still were well aware of options of neutering livestock animals. Pet dogs were typically living outside of homes, often in their dog houses. Gradually there was a movement toward having the dogs inside homes where they came to be considered members of the family (17). As closer contact increased between humans and their dogs, more people began to neuter and spay their dogs. This prevented those dogs from reproducing, and somewhat reduced some of the problematic behaviors of male dogs, especially roaming (18), but some research has found more aggressive problems among neutered dogs (19). Spayed females also have been found to be more aggressive than intact females (19). Anesthetic and surgical techniques for removing the ovaries and uterus of females were sufficiently developed by then that these procedures also were commonplace. Spaying of female dogs became more common in the U.S. than neutering males (20), as is generally the case in many countries today. Spaying the females avoided unplanned or unwanted puppies and eliminated estrous behavior, and the proestrous bleeding of the females which attracted males.

## The Broad Physiological Effects of Neutering From Hormonal Withdrawal

Although neutering is thought of as a means of rendering the male or female infertile by removing the source of sperm and ova, neutering also removes the primary source of testosterone (T) or estrogen (E) which acts on the basal brain hypothalamus and suprachiasmatic nucleus to facilitate some behaviors. People often believe that after neutering, males will no longer have an interest in mating and will be less aggressive toward other male dogs and people. However, these behaviors are mediated by a neural circuitry, and testosterone simply facilitates the neural circuitry. Even with little T available, the male-like behaviors can still be activated in many male dogs (21). In females, the behavioral aspects of estrus are closely tied to E as part of mating coordination, and neutering females largely removes any further display of estrous behavior permanently.

What is less well known is that production of the gonadal hormones is under the control of gonadotropic hormones produced by the pituitary, the luteinizing hormone (LH) and follicle-stimulating hormone (FSH). In intact males, T levels are controlled by means of a feedback mechanism which maintains T at a consistent level. The T production acts on the hypothalamus which produces the gonadotropin releasing hormone (GnRH); if T levels are low, GnRH induces the pituitary to produce more LH until T levels return to normal. The rising LH in an intact male causes more T to be produced, and then LH drops. Following removal of the gonads in males, the production of LH is not suppressed by T working on the feedback loop. LH levels rise—and remain permanently high. A similar process occurs in females; removal of the ovaries results in low estrogen and this causes consistently high levels of LH.

If there are physiological effects of high levels of LH, these could occur wherever there are receptors for LH. Until recently, little was known about this topic. The various LH receptor sites are now better understood, although what functions might be affected remain to be clarified (22). Several LH receptor sites have been identified where possibly LH may activate these sites and cause physiological effects, especially when with high LH levels. The tissue receptor sites are categorized in Table 1, showing potentially associated non-cancerous and cancerous diseases that could be associated with very high levels of LH.

**Table 1:** Receptor sites for the luteinizing hormone in male and female dogs possibly related to non-cancerous and cancerous diseases (22).

**LH receptor sites and possible non-cancerous diseases related to neutering**
*Gastrointestinal tract*—possible increase in appetite
*Urinary tract*, especially in females—urinary incontinence, urinary calculi
*Pancreas*—diabetes mellitus, obesity
*Thyroid gland*— hypothyroidism
*Head of femur*—ligaments and cartilage—hip dysplasia
*Cranial cruciate ligament*—cranial cruciate ligament rupture or tear
*Hippocampus and hypothalamus*—behavioral changes seen with neutering
*Central nervous system*—cognitive decline
**LH receptor sites and possible cancerous diseases related to neutering**
*Prostate gland*—prostate cancer
*Bladder and urethra*—transitional cell carcinoma
*Vascular endothelial and smooth muscle in heart and spleen*—hemangiosarcoma
*Skin*—mast cell tumors
*Lymphoid tissue and lymphocytes*—lymphoma

A perusal of the diseases associated with neutering in some breeds or sexes reveals that the diseases listed in Table 1 can be associated with the tissues which have been identified as receptor sites for LH. This does not mean that LH is causing the disease, but the fact that LH levels reach and sustain very high levels suggests that this factor should be explored. Breeds may differ with regard to specific receptor sites—a point that also could be investigated.

## Implications of Neutering's Effects on Joint Disorders and Cancers for Some Dogs

The data-based findings on 35-breeds of dogs reveal wide differences among breeds with regard to increased risks of joint disorders and/or cancers (23). Given the huge differences in breed sizes and shapes, the differences in vulnerability to joint disorders or cancers associated with ages of neutering are not surprising.

Knowing about increased risks of disabling joint disorders with neutering, and avoiding the increased risks, is important not just in companion canines but also in working dogs used in police and military work, service dogs working in assisting people in wheelchairs, and dogs used in hunting, herding and agility trials. This emphasizes that the age of neutering should be chosen to avoid the vulnerable period which in some breeds is up to 2 years of age. Avoiding increasing the risk of cancer is important for every role that companion dogs have. There is a range of vulnerabilities to cancers associated with neutering, differing among various breeds, just as is true with joint disorders.

The data also reveal that for most breeds, there is no increased risk of joint disorders or cancers associated with neutering. Thus, from the standpoint of these two disease categories there is no generally recommended age for neutering across all breeds where these diseases were not associated with age of neutering. The caregiver and/or veterinarian can make a personal choice after assessing their own context and situation without adversely increasing risks of these diseases. Veterinarians may wish to provide their own guidelines.

For female dogs, the occurrence of mammary cancer is a concern. Some evident differences were found among the 35 breeds in the proportion of females left intact that developed mammary cancer; in some breeds, as many as 6 percent of intact female dogs had mammary cancer, while other breeds were at 0 percent (22). In one study, the median age of onset of this cancer in intact females was 10 years (24), an age which is considerably older than the median age of dogs in the records of cases in the study of 35 breeds, which was 5–6 years. Thus, one would expect the number of cases with mammary cancer to increase as dogs age further. As mentioned in the paper on 35-breeds, when only cases were counted in which the record went beyond 8 years of age, the differences in proportions of intact females with the cancer did not change. The dangers of mammary cancers in females may be overrated. This view is also supported by a published meta-analysis which concluded that the occurrence of mammary cancer was only weakly related to leaving females intact (25).

Two large studies have examined the relationship between neutering and lifespan. One was based on the Veterinary Medical Database of over 40,000 dogs, and another involved over 20,000 dogs from three separate veterinary hospitals in the US. Both studies found that neutered dogs lived significantly longer than those left intact (26, 27). The studies did not analyze the data with respect to age of neutering. Both studies revealed that neutered dogs were much more likely to die of a cancer [especially those cancers subsequently analyzed in the 35-breed paper: (23)]. The intact dogs were more likely than the neutered dogs to die of trauma, such as automobile accidents or infectious disease. Confounding factors to consider include the variability in size of the dogs, since small dogs live longer. If one wants a long-lived dog, one could be drawn toward a small-dog breed. To reduce the risks of a cancer killing the dog prematurely, one could consider selecting a breed with no cancer vulnerability related to neutering. Additionally, one could choose not to neuter the dog, or at least time the age of neutering beyond the period of vulnerability.

An extensive literature documents the relationship between a decline of gonadal hormones with aging in humans and the increased risk of Alzheimer's disease (28–31). These studies on humans raise the issue of the relationship between the standard of early neutering in dogs and later cognitive decline which results in a relatively longer absence of gonadal hormones in the brain than in the human studies. One study, based on the predictable progression of behavioral changes involved in canine cognitive decline in older dogs, found that neutering of males was associated with an accelerated cognitive decline compared with that in intact males (32). The effect of lifetime absence of gonadal hormones, be it testosterone or estrogen, on cognitive activities, is an area that has scarcely been explored for dogs, especially the possibility of breed differences and the degree of lifetime absence of gonadal hormones.

## Dealing with Dogs That Have Not Been Neutered

Although the authors do not necessarily recommend that caregivers completely avoid neutering their male or female dogs, in many cases this is the choice of the caregivers. This may or may not present problems to work out. For those with dogs of large body size the pursuit of females in estrus may present challenges. With large females, managing the proestrus bleeding may require doggy diapers and housing management. These are issues of less concern in small dogs.

The behavior issues for intact males, such as roaming, urine marking in the house or aggression to another family dog or human family member, sometimes can be reduced by neutering. However, research has revealed that neutering may resolve these behaviors only in a minority of neutered dogs (roaming and urine marking, 40 percent; aggression to another family dog or human, 20 percent) (21). Recent studies point to the complexity of aggressive behavior of male dogs and its triggers, showing that aggression can be increased by neutering. Neutered dogs are more likely to be sniffed in their anal region by intact males and were more anxious and insecure (33); they perhaps present a confusing stimulus that leads to aggressive conflict. Another study found that dogs with a lower percentage of lifetime exposure to gonadal hormones were reported to have 8 fearful and 7 aggressive behaviors reported more frequently by their owners (34). The aggressive problem behaviors dealt were directed at strangers, except for one behavior of being approached by an unfamiliar female dog.

For dogs used in service or assistance roles it may not be acceptable to have an intact dog because of the discipline required and the risk of an intact dog being attracted to, or attracting, dogs of the opposite sex. This is usually not an issue with police or military dogs, and intact German Shepherd males and females were found to be more trainable than neutered dogs for police dog narcotics olfaction performance and behavior; males performed better than females (35). For these various working roles, there could have been extensive training involved. The importance of not neutering too early so as to not unnecessarily increase the risk of a disabling joint disorder has been covered. What may be on the horizon is whether in some breeds, early neutering could advance cognitive impairment as the dog ages, impairing the role in service work or police or military work.

## The Paradigm Shift to Personal Choice in the Timing of Neutering

In general, the idea is to replace the long-standing practice of expecting that the puppy should be spayed or neutered before or at 6 months, or in the case of some females, before the first estrus. Others also raise questions about this across-the-board timing that has been favored the past few decades (36). Instead, the new paradigm is for the veterinarian and pet owner, or the pet owner alone, to use the available data-based information to decide on the best age for neutering. As mentioned, the data suggest that for most of the 35 breeds, there is no increased risk of a joint disorder or cancer with neutering at any age. Thus, the decision with these breeds is up to the owner as to the best time for their circumstances.

The above background is intended to provide the veterinarian or the caregiver of the puppy some information with which to decide on the best age for neutering on a case-by-case basis. Generally, one would focus on the breed and sex of the puppy. If the breed is one of the 35 breeds covered in our study (23), this is straight forward. If the breed is not covered, one could look at closely related breeds. Or, using the paper on mixed breeds (37), one could then see if a mixed-breed puppy in question is expected to mature into one of the weight categories with an increased risk of a joint disorder with neutering at an early age. Finally, data available in supplementary files of the 35-breed and mixed breed papers give the actual estimated risks and percentages for each joint disorder and type of cancer with neutering at each age interval. In some instances, the risk may appear low, even if significant, compared with the other factors being considered, and one should choose not to be guided by the risk alone.

Since ancient times, humans have altered the hormonal status of animals and men with castration, making choices as to which individuals would have these procedures. Emphasizing the concept of personal choice, the idea inherent in the paradigm shift is to take each puppy as a separate case and consider all the relevant factors of the living context when deciding upon the age for neutering. As we learn more about some aspects of neutering such as age-related cognitive decline, there may be some additional considerations that may impact some breeds. The idea of a paradigm shift is that additional relevant information may come about and influence decisions made by puppy owners and their veterinarians.

## Data Availability Statement

The original contributions presented in the study are included in the article/supplementary material, further inquiries can be directed to the corresponding author/s.

## Author Contributions

LH and BH: concept, draft, and edits shared equally. Both authors contributed to the article and approved the submitted version.

## Conflict of Interest

The authors declare that the research was conducted in the absence of any commercial or financial relationships that could be construed as a potential conflict of interest.
